# A one-core, four-dimension framework for teaching–management integrated governance in medical universities

**DOI:** 10.3389/fmed.2026.1952099

**Published:** 2026-09-23

**Authors:** Wenbin Tan

**Affiliations:** School of Basic Medical Sciences, Jining Medical University, Jining, China

**Keywords:** digital-intelligent transformation, educational governance, medical education, smart education, systems theory, teaching-management integration

## Abstract

**Background:**

Digital-intelligent transformation is reshaping medical universities, yet teaching and management often remain structurally separated. This paper proposes a theoretically grounded framework for teaching–management integrated governance (TMIG) in medical universities.

**Main body:**

Drawing on governance theory, general systems theory, digital-era governance, and medical-education standards, we synthesise a one-core, four-dimension conceptual model and specify three enabling logics. The model centres on virtue-oriented talent cultivation and comprises value and philosophy, organisational structure, data platform, and operational mechanism; the theoretical, practical, and safeguarding logics explain how digital-intelligent technologies can couple teaching and management into an evidence-based governance loop. The synthesis is methodologically explicit: it draws on a targeted search of PubMed, Web of Science, Scopus, and policy repositories (2014–2026), applies inclusion criteria focused on digital governance and medical education, and retains approximately 30 anchor works.

**Conclusion:**

By treating teaching and management as one governance system, TMIG offers a conceptually grounded path towards the integrated, intelligent, and internationalised digital-education agenda. The analysis is a conceptual synthesis of published work and contains no primary empirical data; the framework is offered as a foundation for later empirical and evaluative research.

## 1 Introduction

Medical education sits at the intersection of two large transformations. The first is the digital-intelligent transformation of higher education, in which artificial intelligence, learning analytics, cloud platforms, and generative models are no longer peripheral teaching aids but infrastructure that reshapes how institutions operate (1). The second is a renewed emphasis on educational governance—the ways in which universities coordinate academic, clinical, and administrative actors to secure quality and accountability (2, 3). International organisations have framed these shifts as systemic rather than technical. The OECD (1) describes a digital education ecosystem in which management systems, teaching tools, and human capability co-evolve; UNESCO (4) calls for a human-centred approach to generative AI in education and research; and the WHO (5) positions digital health governance as a precondition for equitable, people-centred systems. For medical universities, this external pressure lands on an already demanding mandate: producing clinically competent, ethically grounded physicians while meeting accreditation and quality-assurance standards such as those of the World Federation for Medical Education (6).

A persistent structural problem complicates that mandate. Teaching and management in medical universities are commonly organised as parallel tracks. Teaching—curriculum design, classroom and clinical instruction, assessment of student performance—is owned by academic departments and affiliated clinical schools. Management—enrolment, records, quality monitoring, resource allocation, compliance—is owned by administrative offices and central IT. Digital tools have been introduced on both sides, but largely in isolation: a learning management system on the teaching side, a student-information system on the management side, each with its own data model and its own stakeholders. The result is the familiar pattern of data silos, duplicated data entry, and decisions made without the evidence the other side holds. This separation is not merely inefficient; it undermines the coherence of the educational experience, because the people who teach and the people who steward the institution's data rarely act on the same picture of the student or the programme.

The existing literature has examined the two sides of this divide far more than the bridge between them. On the teaching side, a rapidly growing body of work maps where artificial intelligence and analytics enter medical education. Gordon et al. (7), in BEME Guide No. 84, synthesise 278 publications and chart AI across admissions, teaching, assessment, and clinical reasoning, while noting that governance and administration remain under-examined. Foundational commentaries by Wartman and Combs (8), van der Niet and Bleakley (9), and Rampton et al. (10) anticipate the pedagogical and ethical stakes of AI in medicine. Civaner et al. (11) document unmet demand for AI literacy among medical educators across Turkey.

On the management side, scholars of higher-education governance discuss digital-era coordination, interoperability, and data governance (12–14). What is missing is a framework that treats teaching and management as one governance system and specifies how digital-intelligent technology can couple them. Recent medical-education contributions begin to close this gap—Zhang and Shi (15) propose a collaborative-governance restructuring of medical talent cultivation across medical schools and teaching hospitals, and Li et al. (16) put forward a three-dimensional ethical governance framework for AI in medical education—but an integrative model built explicitly on governance and systems theory, in which the bridge between teaching and management is the unit of analysis, has not yet been articulated.

This gap matters for both theory and practice. Theoretically, the governance literature and the medical-education literature have developed in parallel, with little cross-reference; governance scholars rarely discuss the curriculum, and medical educators rarely discuss the administrative architecture that enables or constrains it. Practically, without an integrative framework, digital investments fracture into incompatible systems, and institutions struggle to translate abundant data into better decisions. The present paper responds to this gap. Its purpose is to construct a theoretically grounded framework for teaching–management integrated governance (TMIG) in medical universities and to specify the logics through which digital-intelligent transformation can realise it. The paper is a conceptual synthesis; it analyses the published work of others and reports no primary data, consistent with the requirements of the supporting educational-reform project.

The medical-university case is not arbitrary. Medical education is unusually high-stakes and tightly regulated: errors in training propagate into patient care, and accreditation constrains what institutions may change. It is also unusually data-rich, spanning basic-science coursework, clinical clerkships, skills labs, and licensing preparation, each generating records that currently rarely speak to one another. These features make the teaching–management divide both more damaging and more visible than in many other disciplines, and they make medical universities a natural site to develop and test an integrative governance model. The Chinese policy context adds urgency: the “new medical sciences” initiative and the integrated, intelligent, and internationalised (3I) digital-education agenda push institutions to couple teaching and management, yet provide little conceptual guidance on how. TMIG is offered as that guidance.

The contribution is threefold. First, the paper brings governance theory and systems theory to bear on a problem usually discussed in narrower technical or administrative terms, showing that the teaching–management divide is a governance design choice rather than a natural fact. Second, it offers a parsimonious “one-core, four-dimension” model that practitioners can map onto existing structures without abandoning them. Third, it translates the model into five numbered, falsifiable propositions (P1–P5) plus three actionable logics—theoretical, practical, and safeguarding—so that the framework is not only descriptive but operable.

This paper is organised as follows. Section 2 sets out the synthesis method, sources, and theoretical foundations. Section 3 defines the core construct—virtue-oriented talent cultivation—elaborates its four dimensions, and enumerates five testable propositions (P1–P5). Section 4 specifies the three enabling logics. Section 5 examines the key implementation challenges. Section 6 discusses transferability and resource feasibility, acknowledges the limitations, and sets out the future research agenda. Section 7 concludes.

## 2 Theoretical foundations

Four bodies of theory underwrite the framework. Read together, they explain why teaching and management should be governed as one system, and how digital-intelligent technology changes the terms of that governance. None alone is sufficient; their combination is what makes TMIG coherent.

2.0 Synthesis method and sources. This paper is a conceptual synthesis, not a systematic review or meta-analysis; we therefore do not report a PRISMA flow diagram. Sources were assembled through a targeted search of PubMed, Web of Science, Scopus, and policy repositories (UNESCO, OECD, WHO, WFME, and Chinese national policy repositories), covering 2014–2026. Search terms combined governance terms (“educational governance”, “digital-era governance”, “joined-up government”) with medical-education terms (“medical education”, “clinical teaching”, “learning analytics”, “AI in medical education”). Inclusion criteria were peer-reviewed conceptual, empirical, or policy literature explicitly addressing digital governance in higher education or AI/analytics in medical education; editorial commentaries, conference abstracts, and studies limited to a single specialty were excluded except where they theorised a governance mechanism. Approximately 30 anchor works published within this window were retained after iterative screening, supplemented by Chinese-language policy documents for context. The synthesis procedure was iterative: candidate frameworks were mapped against the teaching–management interface, and only those that engaged with both sides were carried forward. This method is consistent with narrative-synthesis approaches used in conceptual medical-education work (7) and with the scope statement accompanying the manuscript. Classic theoretical works cited through backward citation (e.g., Rhodes 1996; Dunleavy et al. 2006; Meadows 2008; Cruess and Cruess 2008) lie outside this 2014–2026 window and are included as foundational references; together with these foundational works and the policy documents, the total cited corpus is 40 works.

### 2.1 Governance theory and the turn to “good governance”

Governance theory describes how organisations coordinate across multiple actors to make and implement collective decisions. The new-governance literature (2) shifts attention from hierarchy to networks and from command to negotiation; the broader tradition (3, 17, 18) treats governance as the patterned coordination of state, market, and civil-society actors around a common problem. The relevance to medical universities is direct: clinical teaching increasingly crosses departmental, hospital, and accreditation boundaries, and no single office owns the full chain from admissions to licensure. Good governance, on this account, is less about control than about keeping actors in productive alignment around a stated end (17, 18).

### 2.2 General systems theory

General systems theory (19–21) treats any organised activity as a set of interacting elements whose behaviour cannot be inferred from any one element in isolation. Teaching and management in a medical university are coupled subsystems: a change in curriculum triggers changes in assessment, accreditation evidence, and resource allocation. The systems view discourages “point solutions” that optimise one subsystem at the expense of others; it points instead towards redesigning the boundary between subsystems so that feedback can travel (21). Applied here, systems theory exposes the teaching–management divide as a boundary the institution has drawn—and that the institution can redraw.

### 2.3 Digital-era governance

Digital-era governance (12, 22) extends new-governance thinking to a context in which data and digital infrastructure are core state capacity, not back-office plumbing. Digital-era governance argues for reintegration of functions that New Public Management (NPM) had separated, achieved through shared data, shared platforms, and shared digital services rather than through managerial hierarchy. EDUCAUSE (23) frames digital transformation as an organisational redesign problem rather than a technology-adoption problem. Williamson (13, 24) documents how datafication and algorithmic governance reconfigure evaluation and decision-making in education, and warns that these reconfigurations can also harden existing inequalities if left uncontested.Reich (25) reinforces this caution from the educational-technology side: his synthesis of why large-scale technologies repeatedly fail to transform teaching and learning shows that integration succeeds or fails on institutional design, not on tools.

### 2.4 Medical-education standards and governance of AI in medicine

The fourth body of literature is the standards and ethics literature for medical education and digital health. The WFME (6) treats curriculum and governance as twin pillars of quality; the WHO (26) sets ethical principles for AI in health; García-Peñalvo (27) proposes an institutional reference framework for managing the dark side of digital transformation in higher-education teaching; and the OECD (28) makes the interoperability case that data collected for one purpose should be reusable for another. Recent work specifically applies governance frames inside medical education: Zhang and Shi (15) propose a collaborative-governance restructuring across medical schools and teaching hospitals; Li et al. (16) propose a three-dimensional ethical governance framework (technological ethics, educational philosophy, medical humanities) for AI in medical education. These are the closest neighbours to TMIG; the present paper differs in making teaching–management integration the unit of analysis rather than either inter-institutional collaboration (15) or ethics-in-AI alone (16).

## 3 Conceptual framework: the one-core, four-dimension model

Synthesising the theories above, we propose a teaching–management integrated governance (TMIG) model. The model has one core and four dimensions (Figure 1; Table 1). The core is virtue-oriented talent cultivation—the unifying aim that prevents integration from collapsing into mere efficiency. The four dimensions are the mutually reinforcing subsystems through which the core is realised. They are not sequential stages but concurrent conditions: weakness in any one degrades the others.

**Figure 1:**
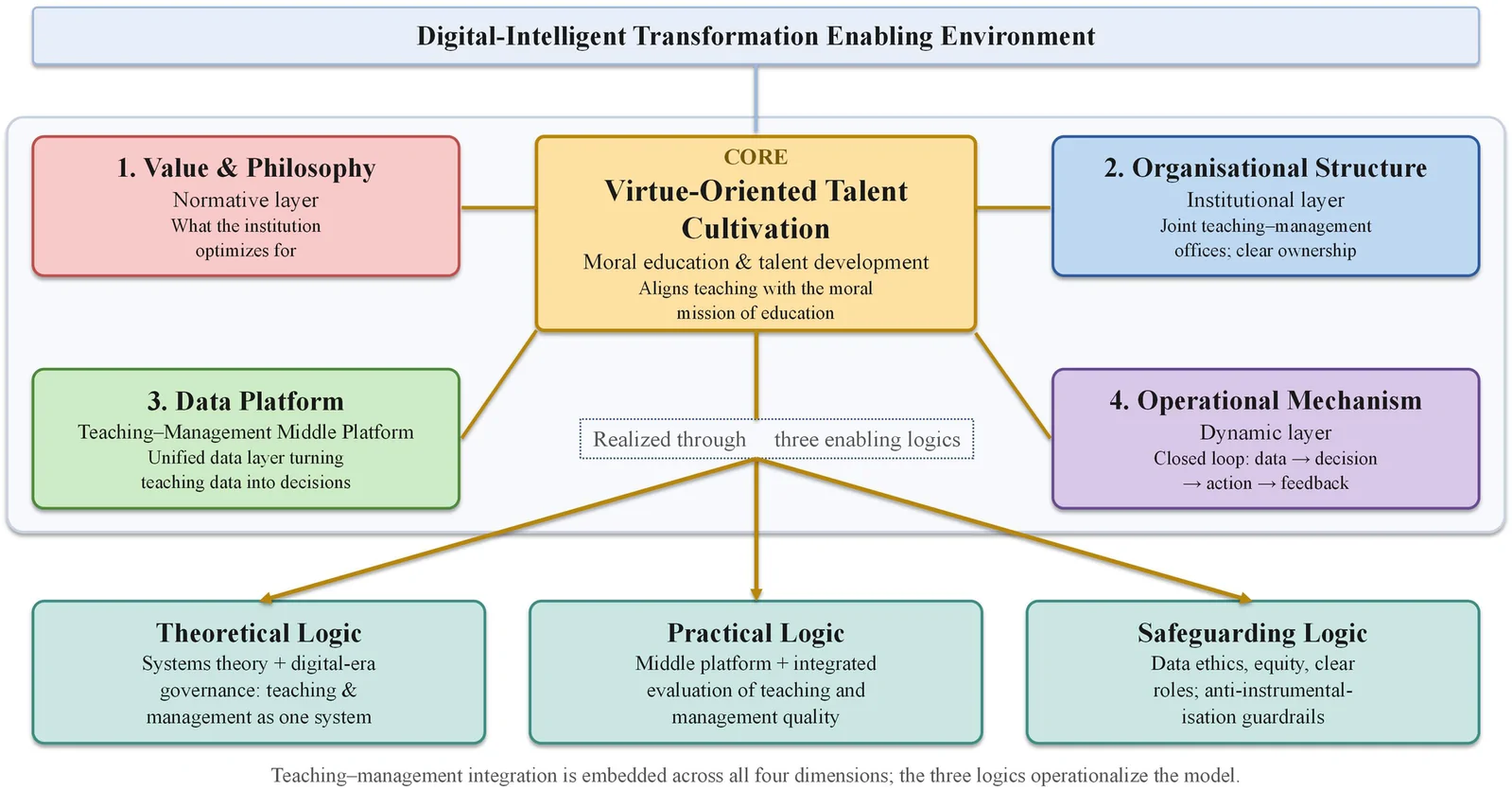
The One-core, four-dimension teaching–management integrated governance (TMIG) model. The core—virtue-oriented talent cultivation—is surrounded by four mutually reinforcing dimensions: value and philosophy, organisational structure, data platform, and operational mechanism. Digital-intelligent transformation supplies the enabling environment; the three logics (theoretical, practical, safeguarding) translate the model into sustained practice.

**Table 1:** The one-core, four-dimension TMIG model. The columns summarise, for each dimension, its core question, principal elements, and integrative mechanism.

Dimension	Core question	Principal elements	Integrative mechanism
Value & philosophy	What are we optimising for?	Human-centred AI; equity; academic mission over metric; ethical use of data	A shared normative compass that subordinates tools to educational purpose
Organisational structure	Who decides, and how are teaching and management linked?	Joint teaching–management committees; clinical–academic co-leadership; cross-functional offices	Architecture that makes integration a responsibility, not a favour
Data platform	Where does shared evidence live?	Educational-management middle platform; interoperable LMS and SIS; learning analytics with consent	A single data spine turning fragmented records into actionable intelligence
Operational mechanism	How does the loop actually turn?	Evidence-based planning; integrated evaluation; feedback and continuous improvement	A closed loop converting data into decisions and decisions into better teaching

### 3.1 Defining the core construct: virtue-oriented talent cultivation

The core refers to the institutional commitment that medical education is, first and last, the formation of virtuous physicians—clinically competent, ethically grounded, and accountable to the public—rather than the production of graduates against measurable proxies. The construct draws on two intersecting traditions: (a) the Chinese policy discourse of “立德树人” (li-de shu-ren, “establish virtue and nurture people”), which anchors recent national reforms of medical education and the 3I digital-education agenda (the codifying policy instruments are cited in Section 6); and (b) the long-standing medical-humanities and virtue-ethics literature that places professional virtue at the centre of medical practice. Internationally, the core maps onto concepts such as professionalism, formation, and the public-good orientation of medicine; in non-Chinese contexts it is therefore best read as a re-statement of the well-established medical-education principle that technical mastery must be subordinated to moral purpose. This re-statement matters because it specifies what the four dimensions are for: each dimension is judged by whether it serves the formation of virtuous physicians, not by whether it improves a given metric.

The four dimensions elaborate the core in operational terms.

Value and philosophy is the normative layer. It asks what the institution is optimising for and refuses the answer “efficiency” on its own. It insists that AI and analytics serve the academic mission, that equity is a design constraint rather than an afterthought, and that data are used ethically and with consent (26). Without this layer, the other three dimensions optimise a goal no one chose.

Organisational structure is the institutional layer. Integration fails when no one owns it, so TMIG calls for joint teaching–management committees, clinical–academic co-leadership of programmes, and cross-functional offices that sit between the dean's academic and administrative arms. This is the concrete realisation of reintegration from digital-era governance (12): it makes coupling a duty assigned to a body, not a courtesy extended between silos.

Data platform is the technical layer. The pivot is an educational-management middle platform that sits between source systems—the learning management system, the student-information system, the clinical-skills and assessment systems—and the people who decide. Rather than each office maintaining its own export, the platform provides a governed, consent-aware view of the learner and the programme. This is where the OECD (28) interoperability argument becomes executable: data collected for one purpose become reusable for another.

Operational mechanism is the dynamic layer. It is the closed loop that converts data into decisions and decisions into better teaching: evidence-based planning at the programme level, integrated evaluation that assesses teaching and management quality with one evidence base, and feedback that drives continuous improvement. A strong platform without this mechanism yields dashboards no one uses; a clear philosophy without it yields slogans no one enacts.

The four dimensions are best read as a system, not a checklist. Value and philosophy set the direction; organisational structure creates the authority to act; the data platform supplies the shared evidence; the operational mechanism closes the loop. A change in one dimension raises the demand on the others—for example, a new data platform immediately exposes whether the organisational structure can actually use it, and whether the value layer has prepared faculty to trust it. This interdependence is why partial reforms disappoint: installing a platform without the committee, or declaring a philosophy without the mechanism, leaves the system unbalanced. TMIG's parsimony is deliberate; it names the minimum set of mutually reinforcing conditions without which integration cannot hold.

### 3.2 Numbered propositions

To satisfy the requirement that the framework generate testable claims, we specify five numbered propositions (P1–P5). Each links a TMIG dimension to a measurable counterpart, providing empirical researchers with hypotheses against which the framework can be examined.

P1 (Organisational structure → coupling). Institutions with formal joint teaching–management committees and shared key performance indicators will exhibit higher interoperability between learning management system (LMS) and student information system (SIS) data—operationalised, for example, as the ratio of automated data flows to manual re-entries—than matched institutions without such committees, holding platform maturity constant.
P2 (Data platform → evidence use). The presence of an educational-management middle platform reduces the mean time between an identifiable at-risk student indicator and an intervention documented in the academic record, compared with institutions relying on office-by-office data exports.
P3 (Operational mechanism → loop integrity). Programmes that implement integrated evaluation of teaching and management quality on a single evidence base will show greater year-over-year stability of their quality-indicator trajectories—that is, smaller fluctuations and fewer abrupt shifts—than programmes that evaluate the two domains on separate evidence bases.
P4 (Value layer → technological purpose). When the value layer makes the formation of virtuous physicians the explicit design criterion for platform features, the rate of faculty-reported purpose displacement (i.e., metrics crowding out learning) will be lower than when platform features are governed by efficiency targets alone.
P5 (Safeguarding logic → equity). Implementation patterns that pair a shared data platform with explicit safeguarding governance (data ethics, algorithmic accountability, faculty digital literacy) will produce smaller digital-divide gradients in student outcomes than patterns that deploy the platform without paired safeguarding, holding baseline connectivity and device access constant.

These propositions are intentionally conservative: each is testable with mixed-methods or quasi-experimental designs and can be falsified by the data. They do not commit the framework to any single technology or vendor; they hold the framework to an institutional design choice that can be observed.

## 4 Logic pathways of digital-intelligent empowerment

A framework is only useful if it explains how to get there. We specify three logics that connect the model to practice (Table 2): the theoretical logic of why integration is feasible, the practical logic of how it is enacted, and the safeguarding logic of how it is sustained. These three logics are not parallel options but the three layers required for the model to leave the page: theoretical (why coupling is feasible), practical (how it is enacted), and safeguarding (how it is kept honest).

**Table 2:** Three enabling logics of digital-intelligent empowerment. The columns summarise, for each logic, its central claim, key enablers, and the primary risk if it is neglected.

Logic	Central claim	Key enablers	Primary risk if neglected
Theoretical	Teaching and management are coupled subsystems; digital technology reduces the cost of coupling	Systems thinking; governance lens; shared ontology of education data	Fragmentation persists; investments fail to cohere
Practical	Integration is enacted through a shared data middle platform and integrated evaluation	Interoperable LMS/SIS; learning analytics; joint decision forums; smart dashboards	Data silos; duplicated effort; decisions without evidence
Safeguarding	Integration endures only with ethics, literacy, and institutional embedding	Data-governance policy; AI ethics review; faculty development; accountability rules	Algorithmic bias; digital divide; purpose lost to procedure

### 4.1 Theoretical logic

The theoretical logic rests on systems theory and digital-era governance. Because teaching and management are coupled subsystems, the marginal cost of linking them falls sharply once a shared data layer exists; digital-intelligent technology is the mechanism that makes coupling cheap enough to be routine. OECD (28) makes the same point from the demand side: the value of digital education appears only when data collected for one purpose can be reused for another. TMIG therefore treats integration not as an aspirational slogan but as the system state that technology now makes economically rational. The theoretical logic also reframes the teaching–management divide as a design variable. If the divide is a boundary we drew, we can redraw it—keeping teaching and management distinct for accountability while coupling them for intelligence. This is the systems-theoretical move of relocating the boundary to serve feedback rather than to block it (21).

### 4.2 Practical logic

The practical logic turns the model into operations. Its pivot is the educational-management middle platform described above. Architecturally, the platform ingests data from the LMS (engagement, submission, performance), the student-information system (enrolment, progression, demographics), the clinical and skills systems (clerkship evaluations, OSCE scores), and quality systems (course evaluations, accreditation metrics), then exposes governed views to authorised roles. Learning analytics built on this base support, rather than replace, human judgement (29, 30). Crucially, analytics must be human-centred: transparent about what they measure, contestable by the students and faculty they describe, and bounded by consent.

Integrated evaluation is the second pillar of the practical logic. It means teaching quality and management quality are assessed with the same evidence base, so that a dip in clerkship performance is visible to both the clinical school and the quality office at the same time, and improvement plans are owned jointly. Front-line cases illustrate the pattern: big data and analytics are reshaping how educators interpret student performance in health professions education (31), and learning analytics applied to virtual patient simulators can trace clinical reasoning in real time (32). These are not yet TMIG in full, but they are the practical logic in embryo.

Sequencing matters. Institutions should not attempt all four dimensions at once. A reasonable order begins with the value layer—securing agreement on what integration is for—then the organisational layer, which assigns ownership, then the data platform, which becomes useful precisely because the first two layers exist to use it, and finally the operational mechanism, matured once evidence begins to flow. Reversing the order—buying a platform first and hoping governance follows—reproduces the silo pattern with newer technology.

### 4.3 Safeguarding logic

The safeguarding logic is what keeps integration from degrading into instrumentation. Three pillars matter. First, data governance: clear rules on collection, consent, security, and access, aligned with WHO (26) and UNESCO and with national privacy law, and codified in institution-level policy rather than left to vendors. Second, algorithmic accountability: bias monitoring and human-over-the-loop review, as urged by Li et al. (16) and the WHO (26) guidance on AI ethics, with audit trails that let decisions be explained and contested. Third, digital literacy and equity: faculty and students must be able to read and contest the systems that read them, and the digital divide must not become an educational divide (33). Without these pillars, integration produces efficient injustice—precise measurement in service of the wrong ends.

## 5 Key challenges

The path is not smooth. Five challenges recur across the literature and warrant explicit attention in any TMIG implementation; each is a place where the model can fail if left unmanaged.

Data silos and interoperability gaps. Many institutions run incompatible systems; OECD (28) reports that in roughly half of surveyed systems learning platforms are not interoperable with student-information systems, forcing manual re-entry and leaving most collected data dormant. TMIG's data platform directly targets this gap, but legacy contracts and fragmented procurement make it slow to close.

### 5.1 Algorithmic bias and ethical drift

AI used in admissions, assessment, or advising can entrench bias and erode human agency (34). An integrated system amplifies both the benefits and the harms of any single model, because its outputs flow into decisions across teaching and management at once. Safeguards must therefore be designed into the platform, not bolted on after harm appears.

### 5.2 Digital divide and equity

Connectivity, device, and capability gaps mean the same platform serves students unequally; Han and Kumwenda (33) argue this must be treated as a health-professions education equity issue, not a technical footnote. Integration that ignores the divide risks widening it, concentrating advantage in already-advantaged learners.

### 5.3 Governance fragmentation and role ambiguity

Integrating teaching and management blurs boundaries between academic and administrative authority; without clear mandates, integration stalls in committee (24, 35). The organisational-structure dimension of TMIG is the designed response, but it requires leaders willing to redistribute authority, which is a political act as much as a technical one.

### 5.4 Data-driven governance critique: audit culture, metric gaming, and academic freedom

The integration TMIG proposes necessarily enlarges the scope of institutional measurement. The critical literature warns that this enlargement is not value-neutral. When a measure becomes a target it ceases to be a good measure [Goodhart's law, summarised in Shore and Wright's (36) recent audit-culture synthesis]; universities have a long history of responding to intensified measurement by teaching to the metric, narrowing what is taught to what is counted, and shifting effort from incommensurable practices to countable ones. Williamson (13, 24) shows how the algorithmic governance of education re-allocates authority from faculty to platform owners and auditors; Reich (25) warns that digital empowerment is partial and can invert means and ends. TMIG's response is structural, not rhetorical: the value-and-philosophy dimension must keep the core construct explicit, the safeguarding logic must include metric-review and an appeal path for those contesting a flag or a rank, and academic-freedom protections must be written into the platform's governance—not negotiated ex post. Faculty development (Section 4.3) is, in this light, a safeguard against metric gaming rather than a one-off training event. Nor is the concern limited to faculty; intensified measurement of student engagement, attendance, and clinical-placement performance through learning analytics introduces parallel risks of student-side surveillance and the datafication of learning, which the safeguarding logic must also address.

## 6 Discussion

How does TMIG differ from adjacent proposals? Gordon et al. (7) map where AI sits in medical education but stop at the level of applications; they chart the territory without redesigning the institution. Tolentino et al. (37) catalogue AI curriculum frameworks for medical students, residents, and practising physicians, showing that content standards are coalescing even as governance standards lag. Zhang and Shi (15) advance collaborative governance of talent cultivation but centre the management restructuring across medical schools and teaching hospitals rather than the teaching–management coupling within one institution. Li et al. (16) supply a three-dimensional ethical governance framework for AI in medical education, indispensable on ethics but silent on the structural integration of teaching and management. TMIG is distinct in two ways. Substantively, it makes the integration of teaching and management its organising principle rather than a by-product. Methodologically, it is a conceptual synthesis grounded in governance and systems theory, stated in numbered propositions (P1–P5) and intended to be tested by later empirical and evaluative work rather than to report such work itself.

The framework also has policy implications at three levels. For ministries and accreditation bodies, TMIG suggests that quality assurance should assess the coupling of teaching and management, not each in isolation—a natural extension of the WFME (6) inclusion of both curriculum and governance among its eight themes. For institutions, it suggests beginning with the data platform and the joint committee before purchasing point solutions, because architecture determines whether later tools compound or conflict. For educators, it reframes digital literacy as a governance competence—the ability to read, contest, and steer the systems that measure one's work—rather than a troubleshooting skill.

Transferability beyond the Chinese policy context. TMIG's terminology reflects the Chinese higher-education reform discourse (3I agenda; “立德树人”) because the case examples and policy framing are drawn from Chinese medical universities. The substantive claims, however, are stated at a level intended to be system-agnostic. The core construct (virtue-oriented talent cultivation) re-states an international principle that medical education is for the formation of virtuous physicians: 立德树人 is codified as the “fundamental task” (根本任务) of education in the CPC Central Committee and State Council's Outline for Building a Strong Education Nation (2024–2035) (38) and embedded in the evaluation-reform mechanisms of the Overall Plan for Deepening Educational Evaluation Reform in the New Era (39), while the same orientation is anticipated in the medical-professionalism tradition, in which Cruess and Cruess (40) ground professionalism in medicine's social contract with society. The construct therefore articulates a cross-cultural commitment to virtuous practice rather than a culturally specific one; the four dimensions describe institutional design choices that any medical school confronts, irrespective of jurisdiction; the three logics derive from governance and systems theory, which are not parochial. Two caveats deserve explicit care. First, the “joint committee” is a functional description, not a naming prescription; in decentralised systems the equivalent is more likely a federated governance arrangement, and in strongly centralised systems a single executive committee. Second, the value layer is the most culture-sensitive of the four dimensions; its content will vary across jurisdictions even if its function does not. Future work should test P1–P5 in jurisdictions with different baseline governance traditions before any claim of global transferability is upgraded from plausible to demonstrated.

Resource implications and feasibility for lower-resourced institutions. The educational-management middle platform is not free. A minimal-viable deployment—identity federation between LMS and SIS, consent-aware ingestion, and a governed analytics view—requires sustained investment in staff, contracts, and integration that is non-trivial for rural, regional, or low- and middle-income country (LMIC) medical schools. TMIG is therefore deliberately sequenced: institutions that cannot resource the full platform should still pursue the value layer and the joint committee (the first two steps of Section 4.2), which cost primarily time and political capital rather than capital expenditure. Federated architectures, shared inter-institutional platforms, and ministry-level shared services are plausible avenues for reducing per-institution cost in LMIC contexts; they also imply that the proposition that “integration requires a single owned platform” is too strong—a deliberately federated architecture is consistent with TMIG so long as the data layer is shared, the safeguards are owned, and the operational mechanism is jointly staffed. This concession narrows but does not abandon the framework; it makes TMIG implementable on a spectrum of resource levels.

Limitations are acknowledged. As a conceptual paper, TMIG has not been empirically validated; its dimensions and propositions are claims to be examined, not findings. The framework is pitched at the institutional level and would need translation to departmental and national scales. The Chinese policy context informs the examples, but the model is stated generally enough to be portable, as discussed above. Future research should operationalise the propositions into measurable indicators, test them across medical universities in different systems and resource contexts, and examine how TMIG interacts with established accreditation cycles. A logical next step is a mixed-methods study linking platform maturity and committee structure to measured improvements in teaching quality and student outcomes.

TMIG is also transferable beyond undergraduate medicine. Nursing, pharmacy, and allied-health programmes face the same teaching–management divide and the same accreditation pressures, and the model's dimensions apply with little modification; only the clinical-placement data sources differ. At the same time, the framework's institutional focus is a boundary, not a blind spot: it does not address system-level coordination across universities or the role of professional bodies and regulators, which future work should examine. A useful next step is a comparative study of medical universities at different maturity levels of digital governance, measuring whether movement along the four dimensions correlates with improvements in teaching quality and student outcomes.

Future research agenda. Five questions follow directly from the framework. First, how should P1–P5 be operationalised into measurable indicators suitable for self-assessment by individual institutions? Second, what institutional configurations best support the joint teaching–management committee across different governance traditions (centralised, federated, network-based)? Third, under what conditions does digital-intelligent integration improve equity rather than merely efficiency—particularly in LMIC and rural settings? Fourth, how can the safeguarding logic be specified as auditable institutional policy that survives leadership turnover? Fifth, how does TMIG interact with established accreditation cycles (WFME, LCME, AMC, GMC) in practice? Answering these would convert TMIG from a conceptual proposal into an evaluable research programme.

## 7 Conclusion

Digital-intelligent transformation gives medical universities a rare opportunity to dissolve the inherited wall between teaching and management. This paper argued that the right response is not more technology on each side but a single governance system in which teaching and management are designed to couple. The proposed one-core, four-dimension model—anchored in virtue-oriented talent cultivation and realised through value, structure, platform, and mechanism—offers a parsimonious language for that redesign. The three enabling logics explain why integration is now feasible, how it is enacted through a shared data platform and integrated evaluation, and how it is kept honest through ethics, literacy, and accountability; the five numbered propositions (P1–P5) make the framework testable rather than merely descriptive.

If medical universities govern teaching and management as one, they turn fragmented data into shared intelligence and fragmented effort into coherent improvement—exactly the capacity the digital-intelligent era demands. The framework is offered not as a finished doctrine but as a foundation other researchers can elaborate, test, and, where warranted, contest. In a period when technology outpaces institution design, that kind of conceptual footing is itself a form of preparedness.

## Data Availability

The original contributions presented in the study are included in the article/Supplementary Material, further inquiries can be directed to the corresponding author.
